# Multifocal Skeletal Muscle Mineralisation Following Severe Trauma and Prolonged Critical Illness: A Case Report

**DOI:** 10.7759/cureus.110306

**Published:** 2026-06-05

**Authors:** Mohammed Uddin, Jawad Naqvi, Sohaib I Uddin, Ayesha Amal

**Affiliations:** 1 Radiology, Stepping Hill Hospital, Stockport NHS Foundation Trust, Stockport, GBR; 2 Radiology, Wythenshawe Hospital, Manchester University NHS Foundation Trust, Wytheshawe, GBR; 3 Acute Medicine, Wrightington, Wigan and Leigh Teaching Hospitals NHS Foundation Trust, Wigan, GBR; 4 General Medicine, Dubai Health, Dubai, ARE

**Keywords:** catabolic state, computed tomography, critical illness-related mineralisation, diffuse skeletal muscle mineralisation, heterotopic ossification, ischaemia-reperfusion injury, metabolic hypercalcaemia, multimodal imaging, post-traumatic calcification, soft tissue calcification

## Abstract

Soft tissue mineralisation is an uncommon complication of severe trauma and critical illness and may occur in association with metabolic derangements, tissue injury and prolonged immobilisation. We present a case of a 26-year-old male patient who developed extensive skeletal muscle calcification following multiple stab wounds, hypovolemic shock, and surgical interventions, including limb amputation and bowel resections. Initial imaging showed no calcification; however, six weeks post-injury, CT demonstrated extensive amorphous calcification in the abdominal wall, chest, iliopsoas, and gluteus maximus. This case highlights the importance of recognising skeletal muscle mineralisation in critically ill trauma patients and the role of multimodal imaging in early diagnosis and management. The aetiology may relate to a catabolic state and disuse, causing osteoclastic bone resorption, demineralisation, and elevated serum calcium with subsequent soft tissue deposition. Soft tissue mineralisation in critically ill trauma patients has been described in association with metabolic derangements and tissue injury. This case also highlights the diagnostic challenges in distinguishing soft tissue calcification, heterotopic ossification, and contrast enhancement in critically ill trauma patients.

## Introduction

Soft tissue mineralisation can be radiologically categorised as calcification or heterotopic ossification (HO) [1-3]. Calcification represents deposition of calcium salts without organised bone, whereas HO denotes mature lamellar bone with cortical and trabecular architecture forming within soft tissues such as muscles, tendons and ligaments [1,2]. HO most commonly occurs after trauma, burns, neurological injury or major orthopaedic procedures and is driven by a combination of local tissue injury, systemic inflammation and osteogenic signalling in mesenchymal progenitor cells [2,4,5].

Soft tissue calcifications are traditionally categorised as dystrophic, metastatic or iatrogenic [3,6]. Metastatic calcification describes calcium deposition in otherwise normal tissues due to altered calcium-phosphate metabolism, typically in the setting of hypercalcaemia and/or hyperphosphataemia [3,6]. In contrast, dystrophic calcification occurs in previously injured or necrotic tissues despite normal serum calcium and phosphate [3]. Metastatic calcification can involve viscera, vasculature, skin and musculoskeletal soft tissues, and may remain asymptomatic or cause functional impairment when peri-articular or muscular involvement is extensive [6].

Major trauma and critical illness are increasingly recognised as states associated with significant disturbances in calcium homeostasis and bone turnover, including phases of hypercalcaemia [7,8]. In otherwise healthy individuals, multifocal muscular mineralisation after major trauma and prolonged critical illness is rarely described [8]. This case report illustrates presumed metastatic soft tissue calcification developing in a young trauma patient with hypercalcaemia and elevated alkaline phosphatase, followed by partial regression after bisphosphonate therapy. By outlining the clinical course, imaging features and metabolic context, we aim to raise awareness of this entity and discuss how early recognition and appropriate imaging can guide conservative management and potentially reduce long-term morbidity [3,6].

## Case presentation

A previously healthy 26‑year‑old male patient was admitted to a tertiary trauma centre with arterial haemorrhage after multiple stab wounds to the chest, right upper limb and right lower limb. On arrival, he developed hypovolaemic circulatory arrest in the Emergency Department and was successfully resuscitated and intubated. An initial contrast‑enhanced whole‑body trauma CT performed on day 0 demonstrated no gross visceral injury or soft tissue mineralisation; however, major arterial injury to the right upper limb necessitated emergent amputation due to critical ischaemia.

On day 3, the patient developed extended-spectrum beta-lactamase (ESBL)‑producing *Escherichia coli* bacteraemia with bowel ischaemia, requiring extended right hemicolectomy with end ileostomy and colostomy mucous fistula. CT at that time showed reduced colonic enhancement, pneumatosis in multiple small bowel loops and portal venous gas, in keeping with extensive ischaemia. Further procedures included mucous fistula excision and multiple re‑look laparotomies, confirming ongoing bowel necrosis and prolonging his intensive care unit stay.

A follow‑up contrast‑enhanced CT of the abdomen and pelvis at approximately three weeks post‑injury (day 21) again showed no definite soft tissue mineralisation, despite ongoing systemic inflammation and immobilisation. Six weeks post‑injury (day 42), repeat contrast‑enhanced CT revealed marked muscular atrophy and multifocal amorphous high‑attenuation foci within the rectus abdominis, abdominal wall musculature, chest wall muscles, iliopsoas and gluteus maximus bilaterally (Figures 1-3). The distribution extended beyond the initial stab wound tracts and was unexpected, given that penetrating injuries were confined to the chest and right‑sided limbs. The attenuation of these foci was within the range typically seen for soft tissue calcification and clearly higher than that of adjacent skeletal muscle.

**Figure 1:**
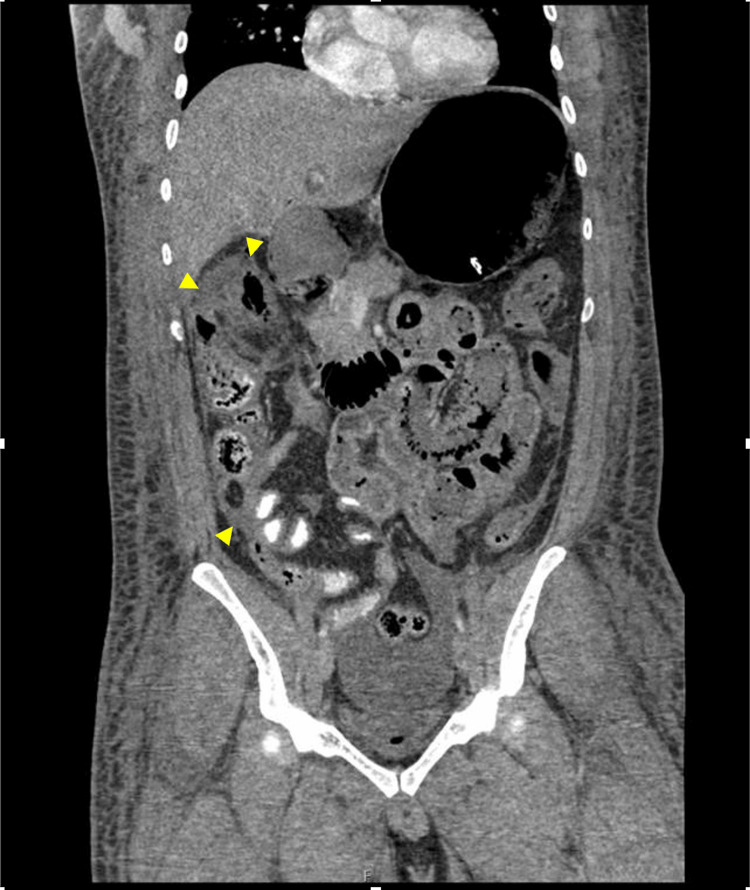
Coronal contrast-enhanced CT (day 0) demonstrating reduced enhancement of the ascending colon (yellow arrowheads), consistent with bowel ischaemia. No abnormal skeletal muscle mineralisation is identified at this stage.

**Figure 2:**
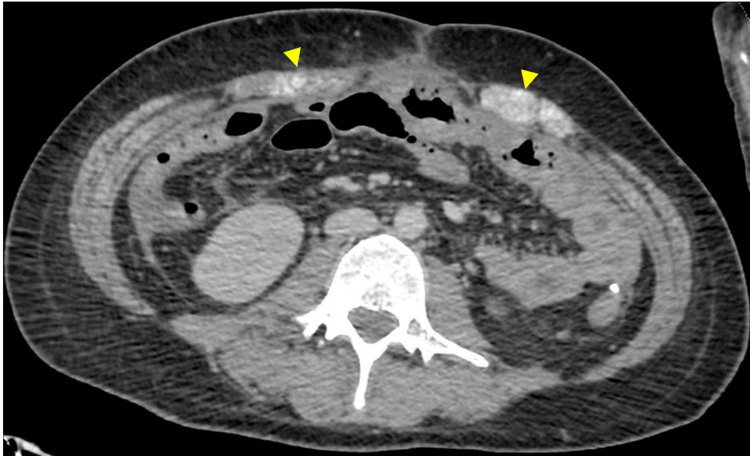
Axial contrast-enhanced CT abdomen demonstrating hyperdense foci within the bilateral rectus abdominis muscles (yellow arrowheads), consistent with soft tissue mineralisation. A midline surgical scar from prior laparotomy is also visible.

**Figure 3:**
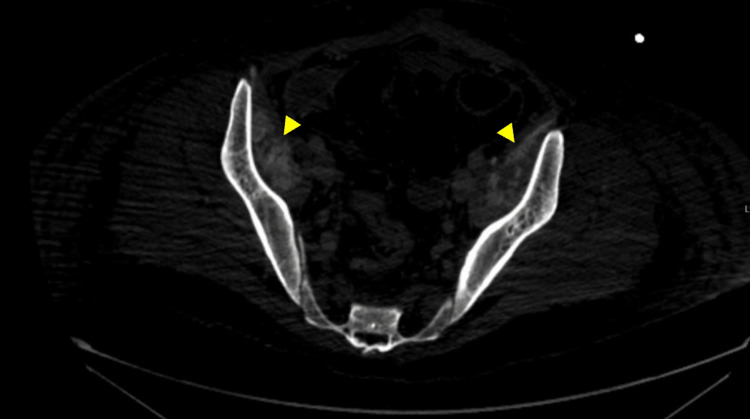
Axial CT bone-windows demonstrating multifocal hyperdense areas within the bilateral iliacus muscles (yellow arrowheads), consistent with skeletal muscle mineralisation. depicting Calcification in bilateral iliacus muscles.

Serum bone profile at six weeks (day 42) demonstrated elevated adjusted calcium and alkaline phosphatase (ALP), with a concomitant increase in serum phosphate. There was no history of chronic kidney disease, known metabolic bone disorder or primary parathyroid disease, and renal function remained within normal limits during admission. Intravenous bisphosphonate therapy was initiated by the metabolic bone team at this stage to treat the hypercalcaemia and address the presumed metabolic drive for soft tissue mineralisation.

Over the subsequent weeks (by day 84), serial blood tests showed normalisation of serum calcium, phosphate and ALP. A follow‑up contrast‑enhanced CT performed several weeks (by day 96) after bisphosphonate therapy demonstrated interval reduction in the size and conspicuity of the muscular high‑attenuation foci, with persistent but less extensive mineralisation in the iliopsoas and abdominal wall muscles (Figure 4). Clinically, as he progressed through rehabilitation, the patient reported generalised stiffness and weakness but no focal joint ankylosis or compressive neuropathy directly attributable to the mineralisation. He remained under follow‑up with the trauma, metabolic bone and rehabilitation teams.

**Figure 4:**
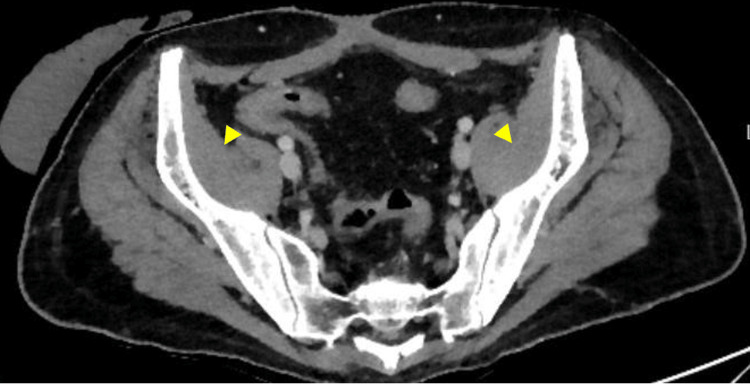
Follow-up axial CT pelvis demonstrating interval resolution of previously identified hyperdense areas within the bilateral iliacus muscles (yellow arrowheads), in keeping with improvement following treatment.

## Discussion

Heterotopic ossification and soft tissue calcification are distinct but sometimes overlapping radiologic entities. Calcification represents relatively homogeneous deposition of calcium salts without organised marrow or cortex, whereas HO comprises mature lamellar bone with cortical and trabecular components forming within soft tissues such as muscles, tendons and ligaments [2,4-7]. HO is well described after trauma, burns, neurological injury and major orthopaedic procedures, driven by a combination of local tissue injury, systemic inflammation and osteogenic signalling in mesenchymal progenitor cells [1,3-5,8-12].

Metastatic calcification, by contrast, relates to systemic disturbances in calcium-phosphate metabolism. Radiologic reviews emphasise that the patterns of dystrophic and metastatic calcification should be interpreted in light of lesion morphology, distribution and biochemical context [13-15]. In our patient, no mineralisation was present on initial trauma CT or on CT at three weeks, but multifocal muscular high-attenuation foci appeared by six weeks, involving the abdominal wall, iliopsoas and gluteus maximus muscles. The delayed onset, multifocal distribution extending beyond the original injury sites and coexistence of hypercalcaemia, elevated ALP and raised phosphate in the absence of chronic kidney disease suggest a multifactorial pathogenesis consistent with metastatic soft tissue calcification in the setting of severe trauma, systemic inflammation and immobilisation.

Severe injury, ischaemic limb loss, bowel ischaemia with sepsis and prolonged intensive care stay likely created a profound catabolic state with accelerated osteoclastic bone resorption, increased circulating calcium and subsequent peri-articular and muscular mineral deposition [7]. Animal models have demonstrated severe injury-induced osteoporosis with subsequent skeletal muscle mineralisation, supporting this mechanism [7]. Large trauma cohorts also report early calcium derangements associated with adverse outcomes, underscoring the clinical relevance of monitoring mineral metabolism in this population [8].

A key diagnostic concern is whether the CT findings simply represent contrast enhancement rather than calcification, given that only contrast-enhanced studies were available. In our case, the foci were amorphous, relatively well-defined areas of high attenuation, with values in the range typically associated with soft tissue calcification and clearly exceeding that of adjacent muscle, and they partially regressed over time in parallel with correction of hypercalcaemia, phosphate and ALP. These features favour true mineral deposition rather than isolated vascular or inflammatory enhancement. Nonetheless, the absence of non-contrast CT, plain radiographs, MRI or scintigraphy means that some diagnostic uncertainty remains, and the possibility of mixed enhancement and mineralisation cannot be entirely excluded. Accordingly, we interpret the findings as presumed metastatic calcification rather than proven heterotopic ossification.

The differential diagnosis for soft tissue mineralisation in this context includes traumatic and post-surgical HO (including myositis ossificans), neurogenic HO after stroke or spinal cord injury, metabolic causes such as calcinosis in renal failure or tumoral calcinosis, crystal deposition diseases and autoimmune connective tissue disorders such as dermatomyositis and scleroderma. Traumatic and neurogenic HO typically demonstrate a zonal maturation pattern with peripheral mineralisation and central lucency on radiographs and CT, subsequently evolving into organised cortical and trabecular bone; this pattern was not observed in our patient [6-10,12-14]. Metabolic calcinosis in renal failure and tumoral calcinosis usually produce large peri-articular masses with amorphous or lobulated calcification and are associated with markedly abnormal renal function, elevated calcium-phosphate product or a positive family history, none of which were present here [14]. Autoimmune calcinosis (calcinosis universalis or circumscripta) is typically associated with a known connective tissue disease and characteristic distribution, which was not seen in this case.

Cross-sectional imaging plays a central role in characterising soft tissue mineralisation. CT is particularly useful because attenuation values help distinguish calcification, typically around 100-400 Hounsfield units, from trabecular and cortical bone, which demonstrate higher densities and internal architecture [6,15]. MRI can further differentiate calcification and early HO, with calcific deposits remaining uniformly hypointense and HO evolving from oedematous, enhancing soft tissue to a mature low-signal rim corresponding to cortical bone [2,5-10]. Bone scintigraphy, especially SPECT/CT, may detect osteoblastic activity before overt mineralisation is visible on CT [3]. Ultrasound and plain radiography can demonstrate superficial or peri-articular deposits and complement CT in equivocal cases. In our patient, the diagnosis relied entirely on contrast-enhanced CT, which is a major limitation.

This case emphasises that in critically ill trauma patients who develop hypercalcaemia and elevated ALP, new multifocal muscular high-attenuation foci on CT should prompt consideration of metastatic soft tissue calcification, even in the absence of pre-existing renal or parathyroid disease. Early recognition is important primarily to guide medical management, optimising calcium and phosphate balance, considering bisphosphonate or other metabolic therapies when appropriate and initiating physiotherapy, rather than to plan surgical excision, which is typically reserved for mature, symptomatic HO [1,4,13]. Increased awareness of this pattern among radiologists, intensivists and surgeons may facilitate earlier metabolic evaluation and follow-up imaging, potentially mitigating progression to more disabling mineralisation or HO [12-15]. Previous studies have described various patterns of soft tissue mineralisation and their imaging characteristics across multiple modalities, including CT, MRI, and nuclear medicine imaging [3,6,15].

## Conclusions

Multifocal skeletal muscle mineralisation may occur in critically ill trauma patients in the context of metabolic disturbance, tissue injury, and prolonged immobilisation. In this case, the clinical, biochemical, and imaging findings were most consistent with presumed metastatic soft tissue calcification; however, definitive differentiation from early heterotopic ossification or contrast-related enhancement was limited by the absence of non-contrast imaging and histopathological confirmation. This case highlights the importance of considering soft tissue mineralisation in the differential diagnosis of new intramuscular hyperdensities on CT in critically ill patients. While recognition of this pattern may assist in guiding metabolic evaluation and clinical management, the findings should be interpreted with caution, and further imaging or correlation may be required where diagnostic uncertainty persists.
